# Further Screening of Entomopathogenic Fungi and Nematodes as Control Agents for *Drosophila suzukii*

**DOI:** 10.3390/insects7020024

**Published:** 2016-06-09

**Authors:** Andrew G. S. Cuthbertson, Neil Audsley

**Affiliations:** Fera, Sand Hutton, York YO41 1LZ, UK; neil.audsley@fera.co.uk

**Keywords:** *Drosophila suzukii*, biocontrol, entomopathogenic nematodes, fungi, integrated pest management

## Abstract

*Drosophila suzukii* populations remain low in the UK. To date, there have been no reports of widespread damage. Previous research demonstrated that various species of entomopathogenic fungi and nematodes could potentially suppress *D.* *suzukii* population development under laboratory trials. However, none of the given species was concluded to be specifically efficient in suppressing *D. suzukii*. Therefore, there is a need to screen further species to determine their efficacy. The following entomopathogenic agents were evaluated for their potential to act as control agents for *D. suzukii*: *Metarhizium anisopliae*; *Isaria fumosorosea*; a non-commercial coded fungal product (Coded B); *Steinernema feltiae*, *S. carpocapsae*, *S. kraussei* and *Heterorhabditis bacteriophora*. The fungi were screened for efficacy against the fly on fruit while the nematodes were evaluated for the potential to be applied as soil drenches targeting larvae and pupal life-stages. All three fungi species screened reduced *D. suzukii* populations developing from infested berries. *Isaria fumosorosea* significantly (*p* < 0.001) reduced population development of *D. suzukii* from infested berries. All nematodes significantly reduced adult emergence from pupal cases compared to the water control. Larvae proved more susceptible to nematode infection. *Heterorhabditis bacteriophora* proved the best from the four nematodes investigated; readily emerging from punctured larvae and causing 95% mortality. The potential of the entomopathogens to suppress *D. suzukii* populations is discussed.

## 1. Introduction

*Drosophila suzukii* Matsumura originates from Southeast Asia being widely distributed in countries such as China, India, Korea, Myanmar and Thailand [1,2,3]. In recent years it has entered many parts of North America [4,5], South America [6] and Europe [7,8] causing severe economic damage [9]. *Drosophila suzukii* was first recorded in the UK in 2012 [10]. Though monitoring traps regularly display its presence across the UK, reports of severe damage have, to date, been restricted to individual fruit farms [11].

*Drosophila suzukii* females pierce with an ovipositor that is heavily sclerotized and serrated the skin of fruits to lay their eggs inside [7,10]. The subsequent larvae then grow and consume the fruit. Larvae will either remain within the fruit or exit it in order to pupate. It has been estimated that upwards of 90% and 93% of *D. suzukii* pupae in raspberry and blueberry crops respectively are found in the soil surrounding the fruit tree base [12]. Therefore, there is potential for soil dwelling or soil applied biological agents to target this life stage [13].

Few studies have investigated biological control of *D. suzukii* [5,14,15]. A range of predatory bugs including *Anthocoris nemoralis*, *Orius majusculus* and *O. laevigatus* have been shown under laboratory conditions to offer potential for being incorporated into integrated control programmes as they feed upon various life stages of *D. suzukii* [14]. Much work has identified several entomopathogenic fungi as important biological control agents of fruit flies [16,17,18,19]. Fungi are able to invade actively through the cuticle of insects which has been cited as an advantage for the management of piercing-sucking insects [20]. Recent studies have investigated several species of entomopathogenic fungi specifically against *D. suzukii* [10,21]. Here, mortality of *D. suzukii* ranged widely between species of fungus screened, with no particular species being determined as offering sufficient control. Direct spray application of fungi onto adult flies did not impact upon them quick enough. The treated flies were still able to produce another generation of flies before they themselves began to die [10].

Entomopathogenic nematodes from the Steinernematidae and Heterorhabditidae are known to be able to penetrate and cause the death of many invertebrate pests of economic importance [22,23] and so form effective alternatives to chemical pesticides [24]. They can be applied, at the infective juvenile (IJ) stage directly with other biological and/or chemical pesticides, fertilizers and soil amendments and it is often more economical to tank-mix nematodes with such inputs for application purposes [25,26]. Nematodes such as *Steinernema feltiae* and *S. carpocapsae*, have been demonstrated to be effective in the control of the quarantine leaf miner species *Liriomyza huidobrensis* on lettuce in commercial glasshouses [27] and also the sweetpotato whitefly, *Bemisia tabaci* [28,29]. Much work has also reported the potential of nematodes against various species of thrips [30,31,32,33] and fruit flies in general [18]. Cuthbertson *et al.* [10] preliminary investigated the use of nematodes specifically against *D. suzukii*. Here, following direct dipping of berries in solutions of Steinernematid nematodes no significant reduction in emergence of *D. suzukii* populations was recorded. However, it was concluded that this was probably not the best application of the nematodes against the fly. The nematodes would be better utilised as ground drenches against larvae/pupae life stages.

The current study investigates a further range of entomopathogenic fungi and nematode species as potential control agents that could be deployed against *D. suzukii*. The fungi were screened against *D. suzukii* life stages occurring on/emerging from the fruit while the nematodes were screened as potential soil drenches targeting larvae/pupae life stages that have fallen from the fruit.

## 2. Materials and Methods

### 2.1. Source of Insects and Control Agents

The *D. suzukii* culture originated from specimens collected from infested fruit from northern Italy [10]. Briefly, collected infested fruit was kept in plastic containers in a controlled environment chamber (25 °C) and emerging flies were collected and identified. They were maintained in bugdorm culture cages on commercial media (*Blades Biological*, Cowden, UK) and supermarket purchased organic blueberries [10]. The entomopathogenic agents used (concentration and supplier) were as follows:

- Fungi: *Metarhizium anisopliae* (1% solution, supplied by Fargo Ltd, Toddington, UK); *Isaria fumosorosea* (1% solution) and a non-commercial coded product (Coded B, 50 mL/L) (both supplied by BASF, Littlehampton, UK).
- Nematodes: *Steinernema feltiae*, *S. carpocapsae*, *S. kraussei* and *Heterorhabditis bacteriophora* (all applied at 10,000 IJ’s/mL supplied by BASF, Littlehampton, UK).

### 2.2. Entomopathogenic Fungi Trials

#### 2.2.1. Treating Infested Berries

Following the method of Cuthbertson *et al.* [10] berries were infested for 72 hours (400 in total) in bug-dorm cages (280 mm × 280 mm × 280 mm; *Watkins and Doncaster*, Leominster, UK) each containing approximately 400 adult mixed-sex flies. One hundred blueberries (10 replicates of 10 berries) were selected at random per treatment. Following full emersion (placing of the berries in the solution for 5 s) in manufacturers’ recommended formulations of the treatment products the berries were incubated in 10 cm diameter plastic deli pots with ventilated lids in a controlled environment (CE) cabinet for 10 days at 25 °C, 65% r.h. and 16:8 L:D. Following this all larvae, pupae and adult flies were counted. Berries were also dissected under a light microscope to fully determine the presence of any larvae. Equal numbers of berries dipped in water acted as controls.

#### 2.2.2. Treating Uninfested Berries

Again, following the method of Cuthbertson *et al.* [10], the potential of the fungi to reduce *D. suzukii* population numbers by treating clean berries and then exposing them to adult flies was investigated. The berries (100 per treatment; 10 replicates of 10 berries) were first dipped in the standard dose rates of the fungal products (as above). They were then placed in 9 cm diameter Petri dishes (10 berries per dish). Berries dipped in water acted as controls. The Petri dishes were then placed into 10 cm diameter plastic deli-pots with ventilated lids. Ten adult *D. suzukii* (5 male and 5 female approximately 10 days old) were then introduced to the berries (while still wet) contained within the deli-pots. All were maintained in a CE cabinet at 25 °C, 65% r.h. and 16:8 L:D. Mortality of the introduced adult flies was assessed over the following week after which all were removed from the berries. The berries were incubated at 25 °C for a total of 10 days after which adult fly emergence was determined. Following this, the berries were dissected and examined for presence of any remaining larvae and/or pupae development.

### 2.3. Entomopathogenic Nematode Trials

#### Potential for Soil Drenching

Following a modified version of the methodology employed by Cuthbertson *et al*. [34], the potential of using the nematodes as soil drenches against *D. suzukii* pupae was investigated. Briefly, 9 cm diameter Petri dishes were filled with 4% v/v of fine commercial children’s play sand (sterilized via heat treatment). Ten *D. suzukii* pupae (approximately 3 days old) were placed on top of the sand. Individual nematode species solution was added containing a concentration of 10,000 IJ/mL over the Petri dish surface. The Petri dishes were placed into 10cm diameter plastic deli-pots with ventilated lids and placed in CE cabinets at 20 °C, 85% r.h. and 6:18 h D:L (dark following application of nematodes [35]). There were 6 replicates for each nematode species (60 *D. suzukii* in total). Pupae on dishes treated with an equal volume of water acted as controls. Dishes were incubated for 14 days and emergence of *D. suzukii* adults recorded. The experiment was repeated by placing *D. suzukii* larvae on sand (approximately 3 days old). The dishes were again incubated for 14 days following which the emergence of adult *D. suzukii* was assessed.

### 2.4. Data Analysis

Data were statistically analyzed as appropriate. Treatments were compared against controls. Assuming normality and constant variance, Analysis of Variance (ANOVA) was used to test for any significant difference.

## 3. Results and Discussion

The current study has added to the knowledge base [10,21] concerning what species of entomopathogenic fungi and nematodes can infect and induce mortality of *D. suzukii*. However, as individual agents, they may not be enough to control fly populations.

**Figure 1:**
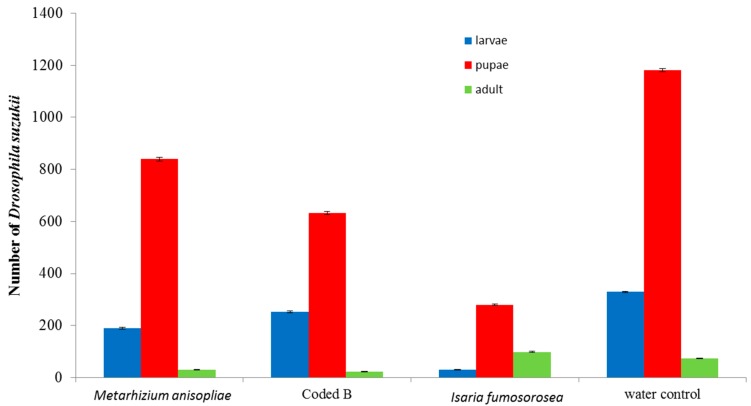
Number of surviving *Drosophila suzukii* life stages from infested berries 10 days after exposure to dip-treatment. Bars are standard errors (±SEM) of the mean.

**Figure 2:**
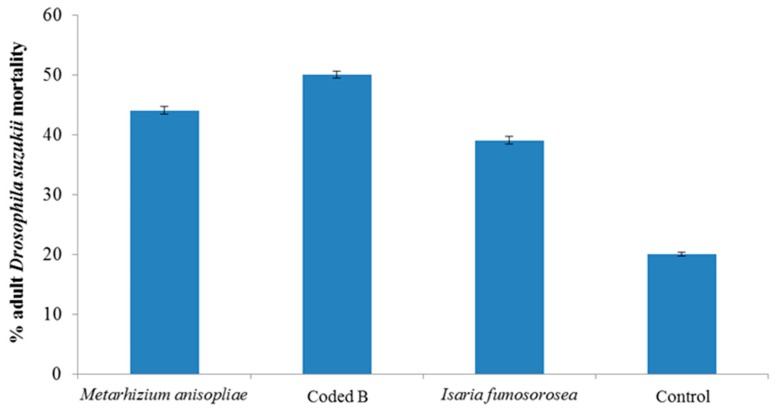
Mortality of adult *Drosophila suzukii* following 7 days exposure to fungi treated berries. Bars are standard errors (±SEM) of the mean.

**Figure 3:**
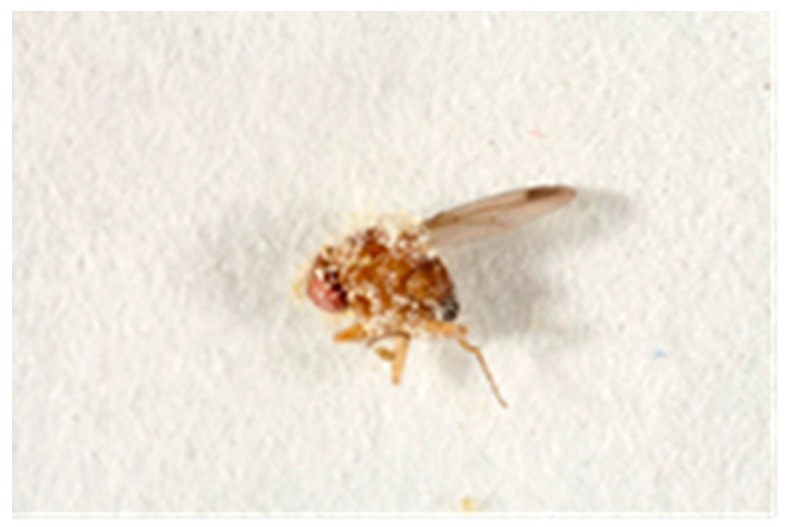
Adult *Drosophila suzukii* infected with entomopathogenic fungi (*Isaria fumosorosea*) (photo: Fera©, Sand Hutton, York, UK).

**Figure 4:**
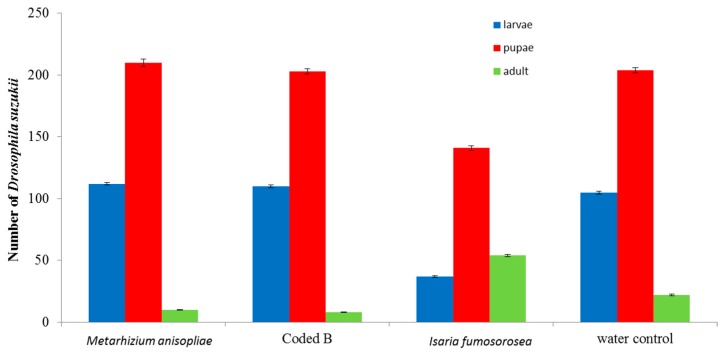
Number of surviving *Drosophila suzukii* life stages 10 days after a 7-day exposure with adult flies to treated berries. Bars are standard errors (±SEM) of the mean.

All three fungi species reduced *D. suzukii* developing in infested berries compared to the water control (*p* < 0.01). *Isaria fumosorosea* significantly (*p* < 0.01) suppressed population development of *D. suzukii* compared to the other fungi species (Figure 1). Following 7 days, mortality of initial adult flies exposed to the berries was recorded (Figure 2). The non-commercial Coded product caused the highest mortality (49%). Fungal growth was noted on adult flies after 7 days (Figure 3). Pre-treating the blueberries with the fungi and then exposing them to adult *D. suzukii* had a lesser impact on resulting population development suppression (Figure 4).

Entomopathogenic fungi, mainly *B. bassiana* and *M. anisopliae*, are known to be highly virulent against the Mediterranean fruit fly and can infect adults, larvae or pupae via different routes of exposure [36,37,38,39,40]. In addition, *I. fumosorosea* has also been shown to be virulent for the Mediterranean fruit fly [36]. Both *M. anisopliae* and, in particular, *I. fumosorosea* proved efficient at suppressing *D. suzukii* population development in the current study. Both fungi also caused ≥40% mortality within 7 days upon contact with adult flies.

**Figure 5:**
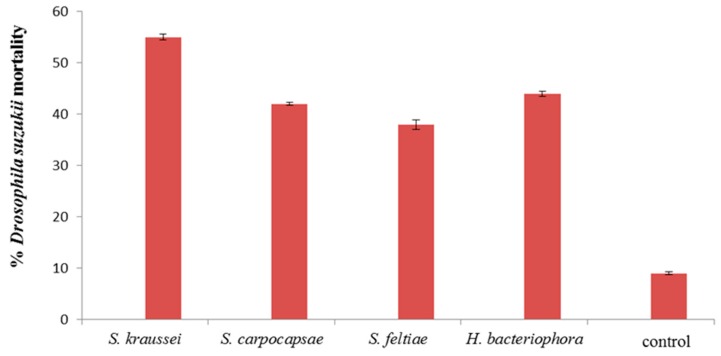
Mortality, at day 14, of *Drosophila suzukii* pupae exposed to 10^4^ IJ/mL nematodes applied as a soil drench. Bars are standard errors (±SEM) of the mean.

The nematodes all had an impact on survival of both pupae and larvae of *D. suzukii*. All nematodes significantly reduced adult emergence from pupal cases compared to the water control (*p* < 0.001) (Figure 5), proving that the nematodes can infect *D. suzukii* pupae. *Steinernema kraussei* caused significantly higher pupal mortality compared to the other nematode species (*p* < 0.05).

**Figure 6:**
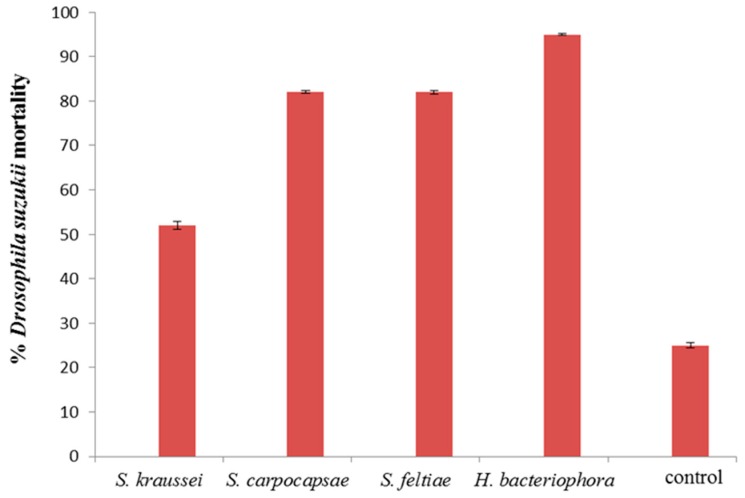
Mortality, at day 14, of *Drosophila suzukii* larvae exposed to 10^4^ IJ/mL nematodes applied as a soil drench. Bars are standard errors (±SEM) of the mean.

**Figure 7:**
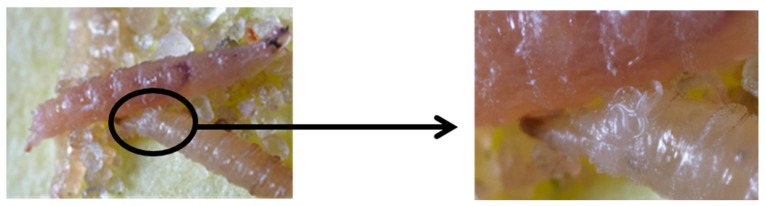
*Heterorhabditis bacteriophora* readily emerging from punctured infested *Drosophila suzukii* larvae (photo: Fera© Sand Hutton, York, UK).

Larvae proved more susceptible to nematode infection (Figure 6). Greater than 50% mortality was achieved by all nematode species. *Heterorhabditis bacteriophora* proved the best from the four nematodes investigated; readily emerging from punctured larvae (Figure 7). Approximately 95% larval mortality was obtained with *H. bacteriophora,* significantly higher than that obtained for the other nematode species (*p* < 0.05). The susceptibility of insects to nematode control agents generally declines with increases in insect size. This has been demonstrated with mermithid nematodes against mosquito larvae [41] and *Bemisia tabaci* larvae [29,42]. Only one *D. suzukii* larval stage (2nd instar) was investigated in this study; perhaps younger instar larvae of *D. suzukii* may be even more susceptible. The nematode induced mortalities in this study are much higher than those obtained by Woltz *et al*. [43] who stated that they found little evidence supporting the use of nematodes against *D. suzukii*. This may simply be due to the higher concentration of nematodes used in the current study. Woltz *et al.* [43] stated that neither *H. bacteriophora* nor *S. carpocapsae* were found to infect *D. suzukii* larvae or pupae in either blueberries or diet media; similar to what Cuthbertson *et al.* [10] determined. Again, demonstrating that the nematodes do not work well on being applied to fruit, and as shown from the current study, they perform much better against larvae and pupae when applied as a soil drench.

The development of an integrated pest management strategy using the nematodes and fungi offers one alternative to complete reliance on chemical products for the control of *D. suzukii*. However, information on their respective compatibility with chemicals routinely used against *D. suzukii* is essential in order to offer complete control of the fly. Further work is necessary to fully determine their potential to be incorporated into a management programme.

## 4. Conclusions

*Drosophila suzukii* is not only a threat to the UK but the worldwide soft fruit industry. All potential control/eradication methods and components must be fully evaluated. The biological agents (fungi and nematodes) investigated all caused reductions in population numbers of *D. suzukii*. In particular, both the fungus *I. fumosorosea* and the nematode *H. bacteriophora* offer much potential to be incorporated into control strategies to be employed against *D. suzukii*. However, although they should prove easy to incorporate into existing invertebrate control programmes, as shown for previous entomopathogens in other pest control strategies [44], individually they are unlikely to control/eradicate populations.

## References

[1] Toda M.J. (1987). Vertical microdistribution of Drosophilidae (Diptera) within various forests in Hokkaido. III. The Tomakomai Experiment Forest, Hokkaido University. Res. Bull. Coll. Exper. For..

[2] Oku T. (2003). SWD: Drosophila suzukii (Matsumura) in Japan. Agricultural Pest Encyclopedia.

[3] Hauser M., Gaimari S., Damus M. Drosophila suzukii new to North America. North American Dipterists Society.

[4] Lee J.C., Bruck D.J., Dreves A.J., Ioriatti C., Vogt H., Baufeld P. (2011). In focus: Spotted wing drosophila, *Drosophila suzukii*, across perspectives. Pest Manag. Sci..

[5] Walsh D.B., Bolda M.P., Goodhue R.E., Dreves A.J., Lee J., Bruck D.J., Walton V.M., O’Neal S.D., Zalom F.G. (2011). *Drosophila suzukii* (Diptera: Drosophilidae): Invasive pest of ripening soft fruit expanding its geographic range and damage potential. J. Integr. Pest. Manag..

[6] Deprá M., Poppe J.L., Schmitz H.J., De Toni D.C., Valente V.L. (2014). The first records of the invasive pest *Drosophila suzukii* in the South American continent. J. Pest Sci..

[7] Asplen M.K., Anfora G., Biondi A., Choi D-S., Chu D., Daane K.M., Gibert P., Gutierrez A.P., Hoelmer K.A., Hutchison W.D. (2015). Invasion biology of spotted wing drosophila (*Drosophila suzukii*): A global perspective and future priorities. J. Pest Sci..

[8] Calabria G., Máca J., Bächli G., Serra L., Pascual M. (2012). First records of the potential pest species *Drosophila suzukii* (Diptera: Drosophilidae) in Europe. J. Appl. Entomol..

[9] Goodhue R. E., Bolda M., Farnsworth D., Williams J. C., Zalom F. G. (2011). Spotted wing drosophila infestation of California strawberries and raspberries: Economic analysis of potential revenue losses and control costs. Pest Manag. Sci..

[10] Cuthbertson A.G.S., Collins D.A., Blackburn L.F., Audsley N., Bell H.A. (2014). Preliminary screening of potential control products against *Drosophila suzukii*. Insects.

[11] Cuthbertson A.G.S, Audsley N. (2015). Personal Observations.

[12] Woltz J.M., Lee J.C. Biological control of spotted wing drosophila (*Drosophila suzukii*), Poster Presentation. Proceedings of 62nd American Entomological Society Congress.

[13] Renkema J.M., Telfer Z., Gariepy T., Hallett R.H. (2015). *Dalotia coriaria* as a predator of *Drosophila suzukii*: Functional responses, reduced fruit infestation and molecular diagnostics. Biol. Control.

[14] Cuthbertson A.G.S., Blackburn L.F., Audsley N. (2014). Efficacy of commercially available invertebrate predators against *Drosophila suzukii*. Insects.

[15] Haye T., Girod P., Cuthbertson A.G.S., Wang X.G., Daane K.M., Hoelmer K.A., Baroffio C., Zhang J.P., Desneux N. (2016). Current SWD IPM tactics and their practical implementation in fruit crops across different regions around the world. J. Pest Sci..

[16] Mar T.T., Lumyong S. (2012). Evaluation of effective entomopathogenic fungi to fruit fly pupa, *Bactrocera* spp. and their antimicrobial activity. Chiang Mai J. Sci..

[17] Elbashir M.I., Bishwajeet P., Shankarganesh K., Gautam R.D., Sharma P. (2014). Pathogenicity of Indian isolates of entomopathogenic fungi against important insect pests and natural enemies. Ind. J. Entomol..

[18] Soliman N.A., Ibrahim A.A., El-Deen M.M., Shams Ramadan N.F., Farag S.R. (2014). Entomopathogenic nematodes and fungi as bioControl agents for the peach fruit fly, *Bactrocera zonata* (Saunders) and the Mediterranean fruit fly, *Ceratitis capitata* (Wiedemann) soil borne-stages. Egypt. J. Biol. Pest Co..

[19] Yousef M., Garrido-Jurado I., Quesada-Moraga E. (2014). One *Metarhizium brunneum* Strain; Two uses to control *Ceratitis capitata* (Diptera: Tephritidae). J. Econ. Entomol..

[20] Poprawski T.J., Greenberg S.M., Ciomperlik M.A. (2000). Effect of host plant on *Beauveria bassiana* and *Paecilomyces fumosoroseus* induced mortality of *Trialeurodes vaporariorum* (Homoptera: Aleyrodidae). Environ. Entomol..

[21] Naranjo-Lazaro J.M., Mellin-Rosas M.A., Gonzalez-Padilla V.D., Sanchez-Gonzalez J.A., Moreno-Carrillo G., Arredondo-Bernal H.C. (2014). Susceptibility of *Drosophila suzukii* Matsumura (Diptera: Drosophilidae) to entomopathogenic fungi. Southwest. Entomol..

[22] Poinar G.O., Gaugler R., Kaya H.K. (1990). JR Biology and taxonomy of Steinernematidae and Heterorhabditidae. Entomopathogenic Nematodes in Biological Control.

[23] Kaya H.K., Gaugler R. (1993). Entomopathogenic nematodes. Annu. Rev. Entomol..

[24] Cross J.V., Solomon M.G., Chandler D., Jarrett P., Richardson P.N., Winstanley D., Bathon H., Huber J., Keller B., Langenbruch G.A. (1999). Biocontrol of pests of apples and pears in Northern and Central Europe: 1. Microbial agents and nematodes. Biocontrol Sci. Techn..

[25] Krishnayya P.V., Grewal P.S. (2002). Effect of neem and selected fungicides on viability and virulence of the entomopathogenic nematode *Steinernema feltiae*. Biocontrol Sci. Techn..

[26] Cuthbertson A.G.S., Head J., Walters K.F.A., Murray A.W.A. (2003). The integrated use of chemical insecticides and the entomopathogenic nematode, *Steinernema feltiae*, for the control of the sweetpotato whitefly, *Bemisia tabaci*. Nematology.

[27] Williams E.C., Walters K.F.A. (2000). Foliar application of the entomopathogenic nematode *Steinernema feltiae* against leafminers on vegetables. Biocontrol Sci. Technol..

[28] Cuthbertson A.G.S., Walters K.F.A., Northing P., Luo W. (2007). Efficacy of the entomopathogenic nematode, *Steinernema feltiae*, against sweetpotato whitefly, *Bemisia tabaci*, under laboratory and glasshouse conditions. Bull. Entomol. Res..

[29] Cuthbertson A.G.S., Mathers J.J., Northing P., Luo W., Walters K.F.A. (2007). The susceptibility of immature stages of *Bemisia tabaci* to infection by the entomopathogenic nematode *Steinernema carpocapsae*. Russ. J. Nematol..

[30] Chyzik R., Glazer O., Klein M. (1996). Virulence and efficacy of different entomopathogenic nematode species against western flower thrips (*Frankliniella occidentalis*). Phytoparasitica.

[31] Ebssa L., Borgemister C., Berndt O., Poehling H.M. (2001). Efficacy of entomopathogenic nematodes against soil-dwelling life stages of western flower thrips, *Frankliniella occidentalis* (Thysanoptera: Thripidae). J. Invertebr. Pathol..

[32] Funderburk J., Stavisky J., Tipping C., Gorbet D., Momol T., Berger R. (2002). Infection of *Frankliniella fusca* (Thysanoptera: Thripidae) in peanut by the parasitic nematode *Thripinema fuscum* (Tylenchidae: Allantonematidae). Environ. Entomol..

[33] Cuthbertson A.G.S., North J.P., Walters K.F.A. (2005). Effect of temperature and host plant leaf morphology on the efficacy of two entomopathogenic biocontrol agents of *Thrips palmi* (Thysanoptera: Thripidae). Bull. Entomol. Res..

[34] Cuthbertson A.G.S., Mathers J.J., Blackburn L.F., Powell M.E., Marris G., Pietravalle S., Brown M.A., Budge G.E. (2012). Screening commercially available entomopathogenic biocontrol agents for the control of *Aethina tumida* (Coleoptera: Nitidulidae) in the UK. Insects.

[35] Cuthbertson A.G.S., Walters K.F.A. (2005). Evaluation of exposure time of *Steinernema feltiae* against second instar *Bemisia tabaci*. Tests Agrochem. Cult..

[36] Castillo M.A., Moya P., Herna´ndez E., Primo-Yu´fera E. (2000). Susceptibility of *Ceratitis capitata* Wiedemann (Diptera: Tephritidae) to entomopathogenic fungi and their extracts. Biol. Control.

[37] Ekesi S., Maniania N.K., Lux S.A. (2002). Mortality in three African Tephritid fruit fly puparia and adults caused by the entomopathogenic fungi, *Metarhizium anisopliae* and *Beauveria bassiana*. Biocontrol Sci. Techn..

[38] Dimbi S., Maniania N.K., Lux S.A., Ekesi S., Mueke J.K. (2003). Pathogenicity of *Metarhizium anisopliae* (Metsch.) Sorokin and *Beauveria bassiana* (Balsamo) Vuillemin, to three adult fruit fly species: *Ceratitis capitata* (Weidemann), *C. rosa* var. *fasciventris* Karsch and *C. cosyra* (Walker) (Diptera: Tephritidae). Mycopathologia.

[39] Konstantopoulou M.A., Mazomenos B.E. (2005). Evaluation of *Beauveria bassiana* and *B. brongniartii* strains and four wild-type fungal species against adults of *Bactrocera oleae* and *Ceratitis capitata*. Biocontrol.

[40] Quesada-Moraga E., Ruiz-García A., Santiago-Álvarez C. (2006). Laboratory evaluation of entomopathogenic fungi *Beauveria bassiana* and *Metarhizium anisopliae* against puparia and adults of *Ceratitis capitata* (Diptera: Tephritidae). J. Econ. Entomol..

[41] Petersen J.J., Willis O.R. (1970). Some factors affecting parasitism by mermithid nematodes in southern house mosquito larvae. J. Econ. Entomol..

[42] Cuthbertson A.G.S., Head J., Walters K.F.A., Gregory S.A. (2003). The efficacy of the entomopathogenic nematode, *Steinernema feltiae*, against the immature stages of *Bemisia tabaci*. J. Invertebr. Pathol..

[43] Woltz J.M., Donahue K.M., Bruck D.J., Lee J.C. (2015). Efficacy of commercially available predators, nematodes and fungal entomopathogens for augmentative control of *Drosophila suzukii*. J. Appl. Entomol..

[44] Cuthbertson A.G.S. (2013). Update on the status of *Bemisia tabaci* in the UK and the use of entomopathogenic fungi within eradication programmes. Insects.

